# Variable Effects of Non-steroidal Anti-inflammatory Drugs (NSAIDs) on Selected Biochemical Processes Mediated by Soil Microorganisms

**DOI:** 10.3389/fmicb.2016.01969

**Published:** 2016-12-05

**Authors:** Mariusz Cycoń, Sławomir Borymski, Bartłomiej Żołnierczyk, Zofia Piotrowska-Seget

**Affiliations:** ^1^Department of Microbiology and Virology, School of Pharmacy with the Division of Laboratory Medicine, Medical University of SilesiaSosnowiec, Poland; ^2^Department of Microbiology, University of SilesiaKatowice, Poland

**Keywords:** NSAIDs, enzyme activities, nitrification and ammonification rate, microbial numbers, soil

## Abstract

Non-steroidal anti-inflammatory drugs (NSAIDs) are the most frequently used group of pharmaceuticals. The high consumption and the uncontrolled disposal of unused drugs into municipal waste or their deposit in landfills can result in an increased concentration of these compounds in soils. Moreover, these drugs can affect the microbial activity. However, there is a lack of knowledge about these effects or it is very limited. Therefore, the objective of this study was to compare the impact of selected commercially available NSAIDs, i.e., diclofenac (DCF), naproxen (NPX), ibuprofen (IBF) and ketoprofen (KTP), applied at concentrations of 1 and 10 mg/kg soil, on the activity of soil microorganisms during the 90-day experiment. To ascertain this impact, substrate-induced respiration (SIR), soil enzyme activities, i.e., dehydrogenase (DHA), acid and alkaline phosphatases (PHOS-H and PHOS-OH) and urease (URE) as well as changes in the rates of nitrification and ammonification processes were determined. In addition, the number of culturable bacteria and fungi were enumerated. In general, the obtained data showed a significant stimulatory effect of NSAIDs on the microbial activity. Higher concentrations of NSAIDs caused a greater effect, which was observed for SIR, PHOS-H, PHOS-OH, URE, N-NO_3_^-^ and N-NH_4_^+^, even during the whole incubation period. Moreover, the number of heterotrophic bacteria and fungi increased significantly during the experiment, which was probably a consequence of the evolution of specific microorganisms that were capable of degrading NSAIDs and used them as an additional source of carbon and energy. However, an inhibitory effect of NPX, IBF or KTP for SIR, DHA, on both phosphatases and culturable bacteria and fungi was observed at the beginning of the experiment. At lower concentrations of NSAIDs, in turn, the effects were negligible or transient. In conclusion, the application of NSAIDs altered the biochemical and microbial activity of soil what may cause the disturbance in soil functioning. It is reasonable to assume that some components of the NSAID formulations could stimulate soil microorganisms, thus resulting in an increase in biochemical activities of the soil.

## Introduction

**FIGURE 1:**
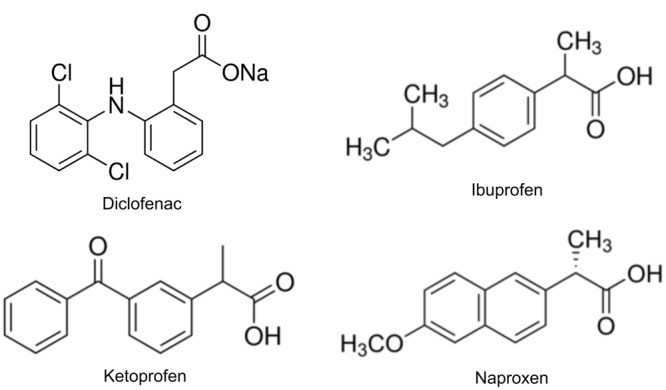
**Chemical structure of the selected non-steroidal anti-inflammatory drugs (NSAIDs)**.

Today, a large amount of different pharmaceuticals are used in human and veterinary medicine in Europe per year. Among them, non-steroidal anti-inflammatory drugs (NSAIDs) are the most frequently used group of pharmaceutical compounds. The use of large quantities of NSAIDs such as diclofenac (DCF) (2-[2-(2,6-dichloroanilino)phenyl]acetic acid), naproxen (NPX) (2*S*-2-(6-methoxynaphthalen-2-yl)propanoic acid), ibuprofen (IBF) (2-[4-(2-methylpropyl) phenyl]propanoic acid), and ketoprofen (KTP) (2-(3-benzoylphenyl)propanoic acid) (**Figure 1**) and their limited removal during wastewater treatment processes has resulted in their increased concentration in the environment (Zorita et al., 2009; Camacho-Muñoz et al., 2012). NSAIDs have been detected in the eﬄuents of wastewater treatment plants, sewage sludge and waters (Tixier et al., 2003; Stackelberg et al., 2004; Weigel et al., 2004; Bragança et al., 2012). Moreover, because of the application of manure, wastewater and sewage sludge in agricultural practice as sources of nutrients for crops, NSAIDs have also been found in soils (Williams and McLain, 2012; Kumirska et al., 2015) and in the runoff from land irrigated with treated wastewater (Pedersen et al., 2005; Topp et al., 2008b; Aznar et al., 2014).

The common occurrence of NSAIDs in the environment is of great concern due to their potential ecotoxicological effect on the aquatic and soil organisms at different trophic levels (Oaks et al., 2004; Schwaiger et al., 2004; Pounds et al., 2008; Gonzalez-Rey and Bebianno, 2014). Moreover, the continuous input of pharmaceuticals into soil, their subsequent accumulation and/or the uncontrolled disposal of unused drugs into municipal wastes or landfills may lead to high NSAIDs concentrations and may pose an unintended risk on living biota (Stuart et al., 2012).

In soil, pharmaceuticals are subjected to physical, chemical, and biochemical processes (Beausse, 2004; Chefetz et al., 2008; Monteiro and Boxall, 2010). However, the fate and persistence of individual NSAIDs in soils depend on a range of factors such as the physicochemical properties of the compound and the characteristics of the environment (Topp et al., 2008a; Monteiro and Boxall, 2009). Based on the water solubility and mobility of pharmaceutical compounds and soil properties, NSAIDs may be adsorbed to soil particles, leached into ground water or runoff into surface water. It has been reported that the leaching of IBF, DCF, NPX, and KTF is significant and is related to their pK_a_ values as well as to the amount of clay and dissolved organic carbon (Xu et al., 2010; Estevez et al., 2014). Studies by Chefetz et al. (2008) proved that both the content and physicochemical nature of soil organic matter (SOM) strongly influenced the sorption of DCF and NPX in SOM-rich soil layers. Pharmaceuticals adsorbed to the organic phase are likely to be far less bioavailable and more persistent than freshly added ones (Al-Rajab et al., 2015). Another mechanism that is responsible for the dissipation of NSAIDs is microbial-mediated degradation (Xu et al., 2009; Carr et al., 2011a,b; Girardi et al., 2013). It has been observed that DCF, IBF and NPX were quickly degraded (0.2–9.5 days) in soil at a concentration of 0.05 mg/kg soil and significantly longer (68 days) when they were applied at a concentration of 5 mg/kg soil (Grossberger et al., 2014). However, environmental factors such as soil type, temperature and moisture strongly affect the degradation rate of these pharmaceuticals (Topp et al., 2008a; Al-Rajab et al., 2010; Carr et al., 2011b; Al-Rajab et al., 2015). Many authors have reported the ability of microorganisms to degrade NSAIDs. A degradative potential has been confirmed for bacterial strains from the genera *Bacillus* (Marchlewicz et al., 2016), *Delftia* (De Gusseme et al., 2011), *Nocardia* (Chen and Rosazza, 1994), *Patulibacter* (Almeida et al., 2013), *Planococcus* (Domaradzka et al., 2015), *Pseudomonas* (Ahmed et al., 2001; De Gusseme et al., 2011; Zhang et al., 2013), *Rhodococcus* (Ivshina et al., 2006), *Sphingomonas* (Murdoch and Hay, 2015), *Stenotrophomonas* (Zhang et al., 2013; Wojcieszyńska et al., 2014), *Variovorax* (Murdoch and Hay, 2015) and fungal strains from the genera *Bjerkandera* (Rodarte-Morales et al., 2012), *Cunninghamella* (Zhong et al., 2003), *Ganoderma, Irpex* (Marco-Urrea et al., 2010c), *Phanerochaete* (Hata et al., 2010; Rodarte-Morales et al., 2012), and *Trametes versicolor* (Marco-Urrea et al., 2010a,b,c; Rodríguez-Rodríguez et al., 2010).

In addition to the degradation of contaminants, soil microorganisms play a key role in the biogeochemical cycling of nutrients and are thought to be sensitive markers of adverse changes in the environment. Thus, any alterations in their abundance and biochemical activity may provide valuable information for the sustainable management and assessment of the potential risks that may be related to the occurrence of pharmaceuticals in soils. There is still little information about the effect of NSAIDs on the microbiological properties of soil exposed to these drugs. Therefore, the objective of the present study was to assess the impact of selected NSAIDs, i.e., DCF, NPX, IBF and KTP, applied at concentrations of 1 and 10 mg/kg soil, on the activity of soil microorganisms during the 90-day experiment. To ascertain this impact, substrate-induced respiration (SIR); soil enzyme activities, i.e., dehydrogenase (DHA); acid and alkaline phosphatases (PHOS-H and PHOS-OH) and urease (URE) as well as changes in the rates of the nitrification and ammonification processes were determined. In addition, the numbers of culturable bacteria and fungi were enumerated.

## Materials and Methods

### Chemicals and Media

Four different commercially available NSAIDs, i.e., Diclac^®^ Duo 150 (diclofenac sodium salt 150 mg – DCF and additional ingredients, i.e., lactose monohydrate, calcium hydrogen phosphate dihydrate, microcrystalline cellulose, magnesium stearate, carboxymethyl starch sodium salt Type A, silica colloidal anhydrous, corn starch, iron oxide E172, and hypromellose), Ketonal^®^ forte (ketoprofen 100 mg – KTP and additional ingredients, i.e., starch, povidone, magnesium stearate, silica colloidal, talc, lactose, hypromellose, macrogol 400, indigotine E132, titanium dioxide, and Carnauba wax), Ibuprom (ibuprofen 200 mg – IBF and additional ingredients, i.e., cellulose, corn starch, guar gum, talc, crospovidone Type A, silica colloidal hydrate, hydrogenated vegetable oil, hydroxypropyl cellulose, macrogol 400, gelatin, sucrose, caolin, confectioners sugar, calcium carbonate, acacia gum, titanium dioxide E171, Opalux White AS 7000, Carnauba wax, and Opacode Black S-1-17823) and Naproxen (naproxen 500 mg – NPX and additional ingredients, i.e., methyl cellulose, croscarmellose sodium, magnesium stearate, and silica colloidal anhydrous) were used in this study. Any other chemicals or media were of an analytical grade and were purchased from Sigma-Aldrich (Germany) or BTL (Poland). The water stock solutions of NSAIDs were sterilized by filtration through 0.22 μm-pore size Millipore membranes and used to prepare the soil treatments. Tryptic soy broth agar (TSBA: tryptone 17 g, soytone 3 g, glucose 2.5 g, NaCl 5 g, K_2_HPO_4_ 1 g, agar 15 g per liter, pH 7.0) and Rose Bengal-Streptomycin Agar (glucose 10 g, peptone 5 g, K_2_HPO_4_ 1 g, MgSO_4_⋅7H_2_O 0.5 g, Rose Bengal 0.033 g, agar 15 g, streptomycin 30 mg/ml per liter, pH 5.6), respectively, were used to enumerate the culturable bacteria and fungi.

### Characteristics of Soil

Soil that had not been previously used for agricultural purposes was collected from the top layer (0–20 cm) of a grass-covered field that was located in the vicinity of Pszczyna in Upper Silesia in southern Poland (49°59′48″ N, 18°55′14″ E). According to the FAO Soil Classification, the soil was classified as Orthic Luvisol. Based on a texture analysis, the soil was found to be loamy sand (sand 85%, silt 12%, and clay 3%). The main characteristics for the soil were the following: density 1.3 g/cm^3^, pH 6.7, cation exchange capacity 13.2 cmol^+^/kg, water-holding capacity (WHC) 33.3%, C_org_ 1.1%, N_tot_ 0.1% and microbial biomass 698 mg/kg). The physico-chemical properties of the soil were determined according to the ISO standards as previously described by Cycoń et al. (2013).

### Design of Experiment

The experiment had a completely randomized block design with the following treatments: control soil (C), soil with DCF – 1 mg/kg soil, soil with DCF – 10 mg/kg soil, soil with NPX – mg/kg soil, soil with NPX – 10 mg/kg soil, soil with IBF – 1 mg/kg soil, soil with IBF – 10 mg/kg soil, soil with KTP – 1 mg/kg soil and soil with KTP – 10 mg/kg soil. There were three replications of each treatment for each sampling time, which produced a total of 135 pots in the experiment (i.e., nine treatments × three replications × five sampling times). In order to ensure an even distribution of NSAIDs in the soil, the water stock solution of each drug was added to sterile quartz sand (<0.5 mm). After the evaporation of the water in the dark, the mixture of sand (50 g/kg soil) and the appropriate drug was added into the soil portion and thoroughly mixed. The final concentrations were calculated per active substance of each NSAID and reflected the most adverse scenarios associated with the entry of large quantities of drugs into the soil as a result of the uncontrolled disposal of unused drugs into municipal waste or their deposit in landfills. The water content of the soils was adjusted to 50% of the maximum WHC and maintained at this level during the entire experimental period. The pots were incubated in the dark at 22 ± 1°C for 90 days. On days 1, 15, 30, 60 and 90, soil samples were taken in order to determine the biochemical processes (soil respiration, enzyme activities, nitrification and ammonification rates) and to enumerate the culturable microorganisms (heterotrophic bacteria and fungi).

### Soil Respiration

The short-term SIR was measured in order to characterize the potentially active soil microbial biomass. Soil samples (100 g) were mixed with glucose (2 g/kg dry weight soil) and the consumption of oxygen was determined within 12 h after the addition of the glucose using the Sensomat Measurement System (LOVIBOND^®^, Germany). The principle of the operation was based on the measurement of the pressure difference in a closed system. During the respiration, CO_2_ was bound to an absorber (45% KOH) and oxygen consumption resulted in a pressure drop that was proportional to the soil respiration. The quantity of oxygen consumed was estimated according to the calculations recommended by the manufacturer.

### Determination of Enzymatic Activity

Dehydrogenase activity was determined using 2,3,5-triphenyltetrazoliumchloride (TTC) as the substrate (0.5%, 5 mL) and incubating the soil samples (5 g) mixed with a Tris buffer (pH 7.6) (0.1 M, 5 mL) at 25°C for 20 h. The produced triphenyl formazan (TPF) was extracted from the mixture with acetone and measured at 546 nm using a Jenway 6300 spectrophotometer (Jenway, UK). The activity of dehydrogenase was expressed as μg TPF/g/h (Alef, 1995).

Acid (PHOS-H) and alkaline (PHOS-OH) phosphatase activities were determined using *p*-nitrophenyl phosphate as the substrate (0.05 M, 1 mL). Soil samples (1 g) were mixed with a modified universal buffer (MUB) (15 mM, 4 mL) of pH 6.5 and 11 for acid and alkaline phosphatase assays, respectively, and the substrate solution and incubated for 1 h at 37°C. After incubation, CaCl_2_ (0.5 M, 1 mL) and NaOH (0.5 M, 4 mL) were added to stop the reaction and to avoid the coloration caused by organic matter. The extracted *p*-nitrophenol (*p*-NP) was measured at 400 nm using a Jenway 6300 spectrophotometer (Jenway, UK). The activity of PHOS-H and PHOS-OH was expressed as μg *p*-NP/g/h (Tabatabai and Bremner, 1969).

Urease (URE) activity was determined using urea (10%, 10 mL) as a substrate, which was added to wet soil (10 g) supplemented with a citrate buffer (pH 6.7) (20 mL) followed by incubation at 37°C for 5 h. After incubation, the NH_4_^+^ concentrations in the reaction mixtures were determined. The analysis was based on the measurement of the intensity of the blue color yielded during the reaction of ammonium with sodium chlorate (0.9% active Cl_2_) and sodium phenolate (phenol 50%, NaOH 21.6%, 1:1) at 630 nm using a Jenway 6300 spectrophotometer (Jenway, UK). The activity of URE was expressed as mg NH_4_^+^/kg/h (Gianfreda et al., 1994).

### Rates of Nitrification and Ammonification

To characterize the overall activity of the soil microorganisms involved in the mineralization of organic nitrogen sources, the ammonium and nitrate concentrations were determined. Soil samples (10 g) were extracted with 100 mL 0.1% K_2_SO_4_ for 24 h followed by a colorimetric determination of the ions in the filtrates. For ammonium, the intensity of the blue color yielded during the reaction with sodium chlorate (1% active Cl_2_, 0.5 mL) and sodium phenolate (phenol 50%, NaOH 21.6%, 1:1) (1 mL) was measured at 625 nm using a Jenway 6300 spectrophotometer (Jenway, UK). For nitrate, the intensity of the yellow color that resulted from the reaction with phenoldisulfonic acid (25% in conc. H_2_SO_4_, 1.5 mL) was measured at 410 nm. The amounts of ammonium and nitrate ions were estimated by reference to the calibration curves and the blank values obtained and the results were calculated per kilogram dry weight soil (PN-R-04028, 1997).

### Enumeration of Culturable Bacteria and Fungi

Soil samples (10 g) were placed in Erlenmeyer flasks containing 90 ml 0.85% sterile NaCl (pH 7.0) for shaking (130 rpm, 30 min) and preparing the serial dilutions for the plate counts. The total number of culturable bacteria was estimated on a TSBA medium. The inoculated plates were incubated at 27°C for 3 days before the colonies were counted. Viable counts of fungi were performed on Rose Bengal-Streptomycin Agar. The plates were incubated at 22°C and fungal colonies were counted after 7 days. The total numbers of culturable bacteria and fungi were expressed as log cfu (colony forming unit)/g dry soil.

### Statistical Analyses

The obtained data were analyzed using the two- and three-way analysis of variance (ANOVA) to determine the percentage of the variation attributable to the factors being tested. The statistical significance of differences (*P* < 0.05) in the measured data was assessed by a *post hoc* comparison of the means using the least significant differences (LSD) test. The separate principal component analyses (PCAs) were carried out with regard to the data obtained for each sampling day as well as the type of drug. Two-way MANOVA analyses were performed for the PC scores obtained for each PCA. All statistical analyses were performed using the Statistica 12.0 PL software package.

## Results

### Substrate-Induced Respiration

**FIGURE 2:**
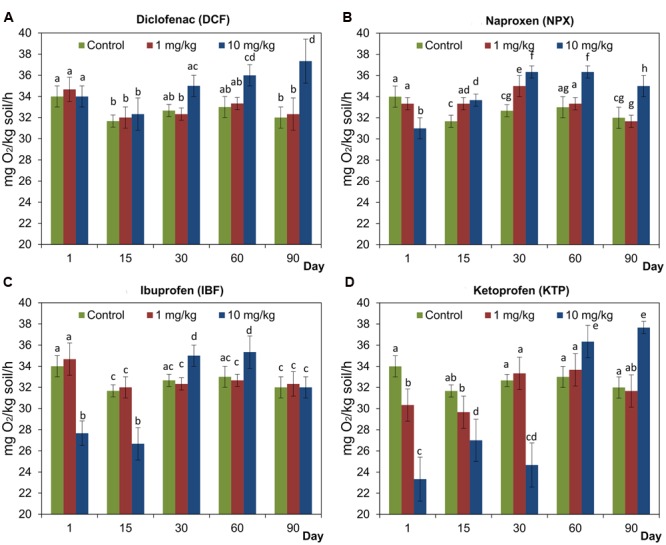
**Substrate-induced respiration (SIR) in the control soil and soil treated with diclofenac (A)**, naproxen **(B)**, ibuprofen **(C)**, and ketoprofen **(D)** at concentrations of 1 and 10 mg/kg soil during the 90-day experiment. The data presented are the means and standard deviations of three replicates and are expressed as mg O_2_/kg soil/h. Different letters (within each drug) indicate significant differences (*P* < 0.005, LSD test) related to the effects of concentration and time.

In general, the obtained data showed a stimulatory effect of NSAIDs on soil respiration. The inhibitory effect of NPX, IBF, or KTP applied at a concentration of 10 mg/kg soil on SIR was observed at the beginning of the experiment. However, the effects were negligible or transient at lower concentrations of NSAIDs (**Figure 2**). The results of a two-way ANOVA showed that the SIR values were significantly affected by the concentration of the drug (*P* < 0.001) in all cases and that this effect explained 7.2–30.4% of the variance. In addition, it was also found that the SIR values were significantly influenced by the time and the interaction between the concentration and the time, which accounted for 19.8–30.5 and 25.1–54.6% of the variance, respectively. The detailed results of a two-way ANOVA analysis are shown in Supplementary Table S1. Taking into account the three factors, the ANOVA indicated that the drug and the time significantly (*P* < 0.001) affected the SIR values for which the observed variabilities were found to be 10.1 and 13.3%, respectively. In addition, significant effects (*P* < 0.001) of all of the possible interactions on the SIR values were found between the factors tested. The lowest value of the variability was observed for the interaction between the drug and the concentration (9.5%), while the highest was observed for the interaction between the concentration and the time (28.6%). The detailed results of a three-way ANOVA analysis are shown in Supplementary Table S2.

### Activity of Dehydrogenase (DHA)

**FIGURE 3:**
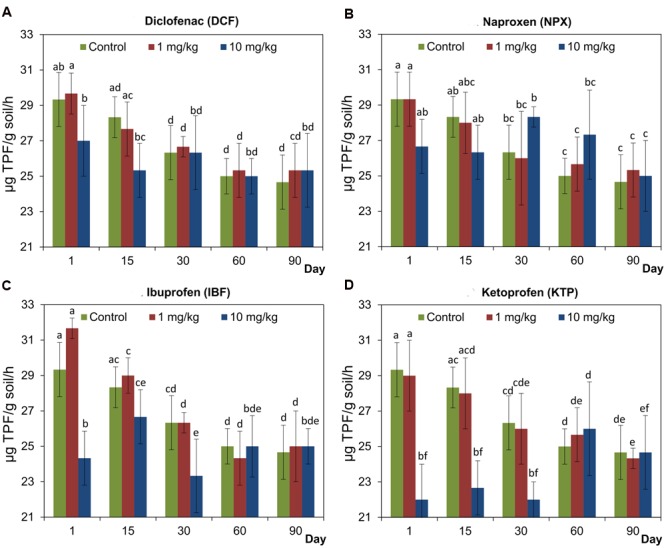
**Dehydrogenase (DHA) activity in control soil and soil treated with diclofenac (A)**, naproxen **(B)**, ibuprofen **(C)**, and ketoprofen **(D)** at concentrations of 1 and 10 mg/kg soil during the 90-day experiment. The data presented are the means and standard deviations of three replicates and are expressed as μg TPF/g soil/h. Different letters (within each drug) indicate significant differences (*P* < 0.005, LSD test) related to the effects of concentration and time.

The study showed a significant effect of NSAIDs on the activity of DHA in soil; however, the observed effect was dependent on the type of drug and its dosage (**Figure 3**). Generally, we found that a higher dose of each drug (10 mg/kg soil) negatively affected the activity of DHA at the beginning of the experiment in all cases. The largest decrease was observed for KTP and this effect was recorded up to day 30 of the experiment. In contrast, no effect was observed in soil treated with a lower dose of NSAIDs (1 mg/kg soil) and the activity of DHA was at the same level as was observed for the control soil (**Figure 3**). The results of a two-way ANOVA showed that the activity of DHA was significantly affected by the time (*P* < 0.001–*P* = 0.037) in all cases and this effect explained 10.4% (KTP)-45.3% (DCF) of the observed variability, respectively. The concentration of drug had a significant effect (*P* < 0.001) only in the cases of IBF and KTP, which accounted for 16.6 and 32.3% of the variance, respectively. It was also found that the interaction between the concentration and the time was not significant with the exception of KTP (*P* < 0.001) for which the observed variability was 30.6%. The detailed results of a two-way ANOVA analysis are shown in Supplementary Table S1. The results of a three-way ANOVA showed that DHA was significantly affected by the drug (*P* = 0.004), the concentration (*P* < 0.001) and the time (*P* < 0.001). The time effect explained most of the variance (25.6%), whereas the concentration accounted for 10.4% of the variance and the drug explained a further 3.4%. In addition, a significant effect (*P* < 0.001) of the interactions between the drug and the concentration as well as the concentration and the time on the activity of DHA was found. The detailed results of a three-way ANOVA analysis are shown in Supplementary Table S2.

### Activity of Acid Phosphatase (PHOS-H)

**FIGURE 4:**
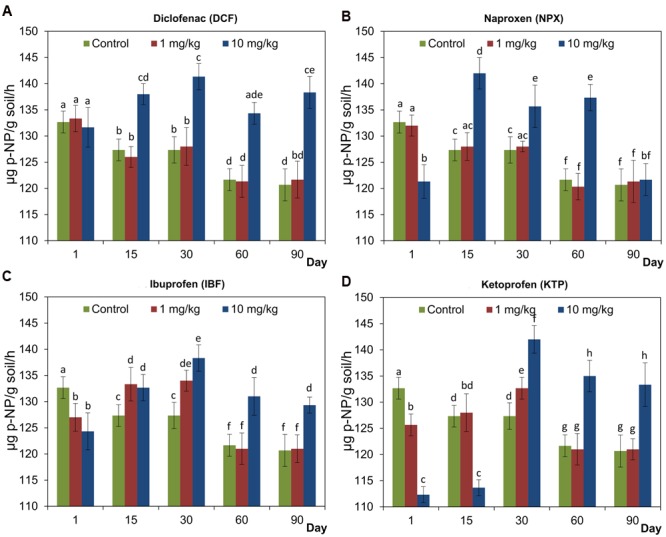
**Acid phosphatase (PHOS-H) activity in control soil and soil treated with diclofenac (A)**, naproxen **(B)**, ibuprofen **(C)**, and ketoprofen **(D)** at concentrations of 1 and 10 mg/kg soil during the 90-day experiment. The data presented are the means and standard deviations of three replicates and are expressed as μg *p*-NP/g soil/h. Different letters (within each drug) indicate significant differences (*P* < 0.005, LSD test) related to the effects of concentration and time.

Generally, all of the NSAIDs applied at a concentration of 10 mg/kg soil positively affected PHOS-H during the incubation period. However, the activity of PHOS-H was significantly inhibited (*P* < 0.05) by NPX, IBF, and KTP at the beginning of the experiment. In turn, at lower concentrations of NSAIDs, the effects were negligible or transient (**Figure 4**). The results of a two-way ANOVA showed that the activity of PHOS-H was significantly (*P* < 0.001) affected by all of the factors tested in all cases. The only exception was the concentration, which had no significant effect on the activity of PHOS-H for KTP. In the case of concentration, the highest observed variability was found for DCF (54.2%) while the lowest was observed for NPX and IBF (14.5%). The time effect explained most of the variance for IBF (40.2%). On the other hand, for KTP up to 68.3% of the observed variability was accounted for by the interaction between the concentration and the time. The detailed results of a two-way ANOVA analysis are shown in Supplementary Table S1. The results of a three-way ANOVA showed that the activity of PHOS-H was significantly (*P* < 0.001) affected by all of the factors tested as well as by all of the possible interactions between these factors. Among the sources of variance, the effect of the interaction between the concentration and the time explained most of the variance (28.4%), whereas the drug effect accounted for only 2.7% of the variance. The detailed results of a three-way ANOVA analysis are shown in Supplementary Table S2.

### Activity of Alkaline Phosphatase (PHOS-OH)

**FIGURE 5:**
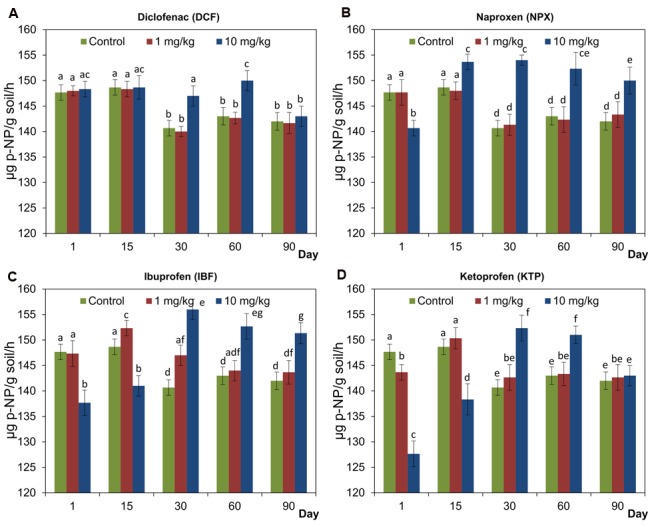
**Alkaline phosphatase (PHOS-OH) activity in control soil and soil treated with diclofenac (A)**, naproxen **(B)**, ibuprofen **(C)**, and ketoprofen **(D)** at concentrations of 1 and 10 mg/kg soil during the 90-day experiment. The data presented are the means and standard deviations of three replicates and are expressed as μg *p*-NP/g soil/h. Different letters (within each drug) indicate significant differences (*P* < 0.005, LSD test) related to the effects of concentration and time.

The study showed a different effect of NSAIDs on the activity of PHOS-OH; however, this effect was more pronounced in the soil treated with higher doses of drugs (10 mg/kg soil) (**Figure 5**). Generally, in all cases, the PHOS-OH was stimulated from day 30 of the experiment. On the other hand, an initial decrease in the activity of this enzyme was observed for NPX, IBF, and KTP. For the first, the activity of PHOS-OH was significantly lower on day 1 after the drug was applied and for the other two, a lower activity of the enzyme was also observed on day 15 of the experiment. However, at lower concentrations of NSAIDs, the effects were negligible (**Figure 5**). The results of a two-way ANOVA showed that the activity of PHOS-OH was significantly affected by all of the factors tested in all cases. The concentration effect explained most of the variance (29.8%) for NPX, while the lowest (2.4%) was observed for KTP. The time effect had the greatest impact on DCF and it accounted for 52.7% of the variance. On the other hand, up to 77.4 and 74.3% of the observed variability was accounted for by the interaction between the concentration and the time for IBF and KTP, respectively. The detailed results of a two-way ANOVA analysis are shown in Supplementary Table S1. Taking into account the three factors, the ANOVA indicated that the drug, the concentration and the time significantly (*P* < 0.001) affected the activity of PHOS-OH for which the variabilities were 4.1, 4.4, and 7.6%, respectively. In addition, significant effects (*P* < 0.001) of all of the possible interactions between the factors tested on the activity of PHOS-OH were found. The highest value of the variability (45.1%) was observed for the interaction between the concentration and the time. The detailed results of a three-way ANOVA analysis are shown in Supplementary Table S2.

### Activity of Urease (URE)

**FIGURE 6:**
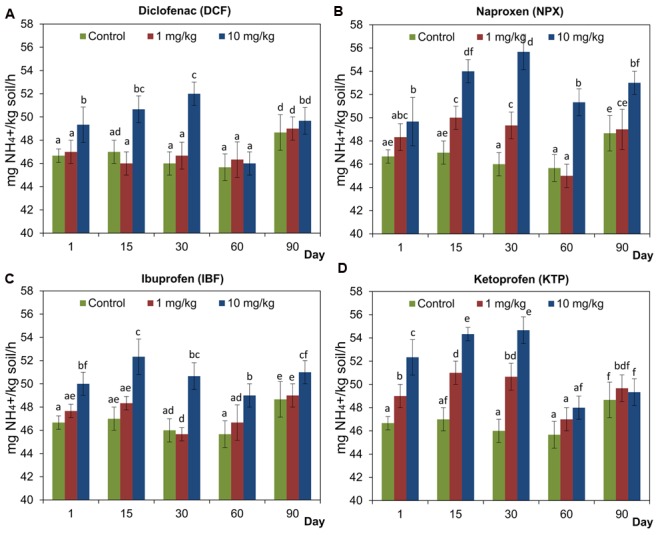
**Urease (URE) activity in control soil and soil treated with diclofenac (A)**, naproxen **(B)**, ibuprofen **(C)**, and ketoprofen **(D)** at concentrations of 1 and 10 mg/kg soil during the 90-day experiment. The data presented are the means and standard deviations of three replicates and are expressed as mg NH_4_^+^/kg soil/h. Different letters (within each drug) indicate significant differences (*P* < 0.005, LSD test) related to the effects of concentration and time.

The analysis of urease activity in the soil treated with selected NSAIDs generally showed that the activity of this enzyme was not inhibited (**Figure 6**). At lower doses (1 mg/kg soil) of NPX and KTP the stimulation of URE activity was observed after 15 and 30 days and after 1, 15, and 30 days, respectively. On the other hand, DCF and IBF used at lower doses did not affect the URE activity throughout the experimental period (**Figure 6**). By contrast, NSAIDs at higher doses (10 mg/kg soil) stimulated the activity of URE. For DCF and KTP, increased activity of URE was observed until day 30, while NPX and IBF stimulated the activity of URE during the 90-day experiment (**Figure 6**). The results of a two-way ANOVA showed that the activity of URE was significantly affected by all the factors tested in all cases. Among the sources of variance, the concentration effect explained most of the variance (34.5% for DCF-62% for NPX). The only exception was the interaction between the concentration and the time, which had no significant impact (*P* = 0.175) for IBF. For the time effect, the lowest value of the variability was observed for NPX (15.7%), while the highest was observed for DCF (23%). The detailed results of a two-way ANOVA analysis are shown in Supplementary Table S1. Taking into account the three factors, the ANOVA indicated that the drug (*P* < 0.001), the concentration (*P* < 0.001) and the time (*P* < 0.001) significantly affected the activity of URE for which variabilities were 6, 45.3, and 14.1%, respectively. In addition, significant effects of all of the possible interactions on the activity of URE were found between the factors tested, which accounted for 4.3–8.7% of the variance. The detailed results of a three-way ANOVA analysis are shown in Supplementary Table S2.

### Rate of Nitrification

**FIGURE 7:**
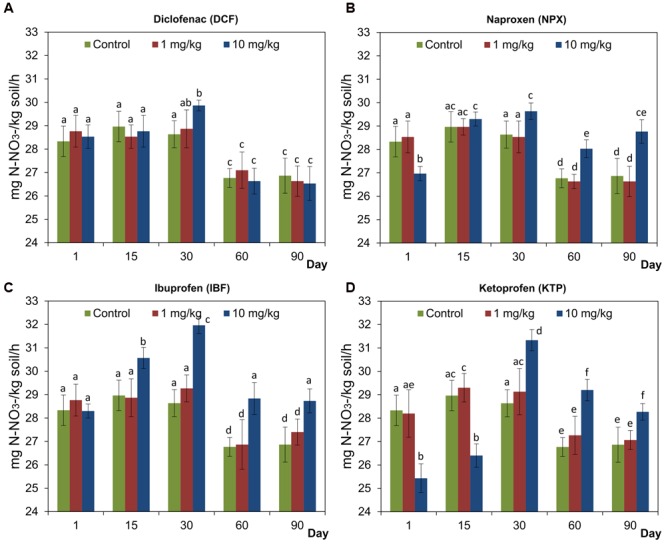
**Concentration of N-NO_3_^-^ in control soil and soil treated with diclofenac (A)**, naproxen **(B)**, ibuprofen **(C)**, and ketoprofen **(D)** at concentrations of 1 and 10 mg/kg soil during the 90-day experiment. The data presented are the means and standard deviations of three replicates and are expressed as mg N-NO_3_^-^/kg soil. Different letters (within each drug) indicate significant differences (*P* < 0.005, LSD test) related to the effects of concentration and time.

The results showed that the rate of nitrification was not affected by significant changes in soil treated with lower doses of any of the NSAIDs compared to the control soil (**Figure 7**). However, there were some differences between the soil treated with higher doses of NSAIDs (10 mg/kg soil) and the control soil, which consisted of a shorter or longer stimulation of nitrification as evidenced by a higher concentration of N-NO_3_^-^ (**Figure 7**). For the soil treated with DCF, the concentration of N-NO_3_^-^ increased only on day 30 after the drug was applied, whereas for IBF this effect lasted from day 15 until the end of the experiment. Higher doses of NPX and KTP also stimulated the nitrification process from day 30; however, a significant decrease in the concentration of N-NO_3_^-^ after application of both drugs was observed at the beginning of the experiment (**Figure 7**). The results of a two-way ANOVA showed that the concentration of a drug had a significant effect on the amount of N-NO_3_^-^ only for NPX (*P* = 0.002) and IBF (*P* < 0.001), which accounted for 7.9 and 28.7% of the variance, respectively. However, the time effect was a significant proportion in the observed variations. The highest value of variability (75.3%) was observed for DCF while in the other cases, the time factor explained 33–50.4% of the variance. The interaction between the concentration and the time also had significant role in the observed variability. For KTP, it was at a level of up to 54.3%. The detailed results of a two-way ANOVA analysis are shown in Supplementary Table S1. The ANOVA including the three factors tested showed a significant effect of the drug (*P* < 0.001), concentration (*P* < 0.001), and time (*P* < 0.001) on the nitrification process. Analyzing the percentage of variation, the largest contribution was observed for the time effect (40.2%), while the drug and concentration effects explained only 3.3 and 4.9% of the variance, respectively. In addition, significant effects of all of the possible interactions between the factors tested on the amount of N-NO_3_^-^ were found, for which the highest value of the variability (15.4%) was observed for the interaction between the concentration and the time. The detailed results of a three-way ANOVA analysis are shown in Supplementary Table S2.

### Rate of Ammonification

**FIGURE 8:**
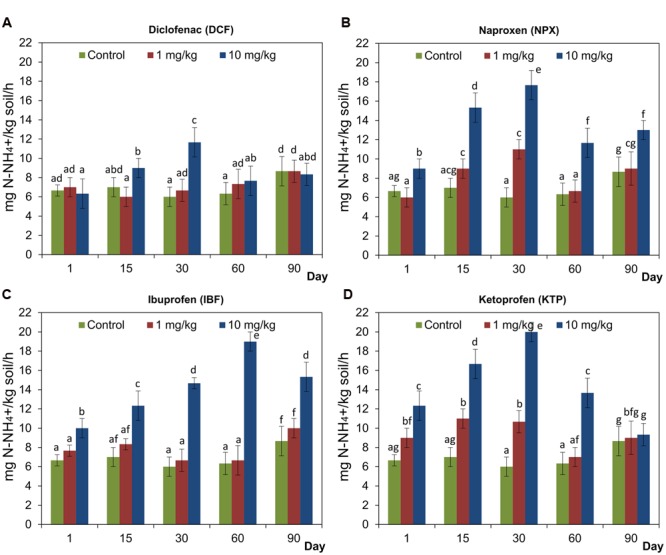
**Concentration of N-NH_4_^+^ in control soil and soil treated with diclofenac (A)**, naproxen **(B)**, ibuprofen **(C)**, and ketoprofen **(D)** at concentrations of 1 and 10 mg/kg soil during the 90-day experiment. The data presented are the means and standard deviations of three replicates and are expressed as mg N-NH_4_^+^/kg soil. Different letters (within each drug) indicate significant differences (*P* < 0.005, LSD test) related to the effects of concentration and time.

The analysis of the concentration of N-NH_4_^+^ in soil treated with selected NSAIDs generally showed that the rate of ammonification was not inhibited (**Figure 8**). At lower doses (1 mg/kg soil) of NPX and KTP, the stimulation of ammonification process after 30 days and after 1, 15, and 30 days, respectively, was observed. On the other hand, DCF and IBF applied at lower doses had no effect on the rate of ammonification throughout the whole experimental period (**Figure 8**). In contrast, all of the NSAIDs used in higher doses (10 mg/kg soil) stimulated ammonification. In the soil treated with DCF, the concentration of N-NH_4_^+^ increased only on day 30 after the drug was applied, whereas for KTP this effect was observed up to 60 days and for NPX and IBF, it lasted the throughout the 90-day experiment (**Figure 8**). The results of a two-way ANOVA showed that the rate of ammonification was significantly affected (*P* < 0.001) by all the factors tested in all cases. Among the sources of variance, the effect of concentration explained most of the variance for NPX, IBF, and KTP (58.6, 69.5, and 60.1%, respectively). Although, all of the factors had a significant effect on the rate of ammonification for DCF, the highest contribution in the observed variability at a level of 35.8% was observed for the interaction between the concentration and the time. The detailed results of a two-way ANOVA analysis are shown in Supplementary Table S1. Taking into account the three factors, the ANOVA indicated that the drug (*P* < 0.001), the concentration (*P* < 0.001) and the time (*P* < 0.001) significantly affected the rate of ammonification for which the observed variabilities were 7.9, 46.9, and 5.7%, respectively. In addition, a significant effect of all of the possible interactions between the factors tested on the concentration of N-NH_4_^+^ was found, which accounted for 6.3–10.6% of the variance. The detailed results of a three-way ANOVA analysis are shown in Supplementary Table S2.

### Number of Culturable Bacteria

**FIGURE 9:**
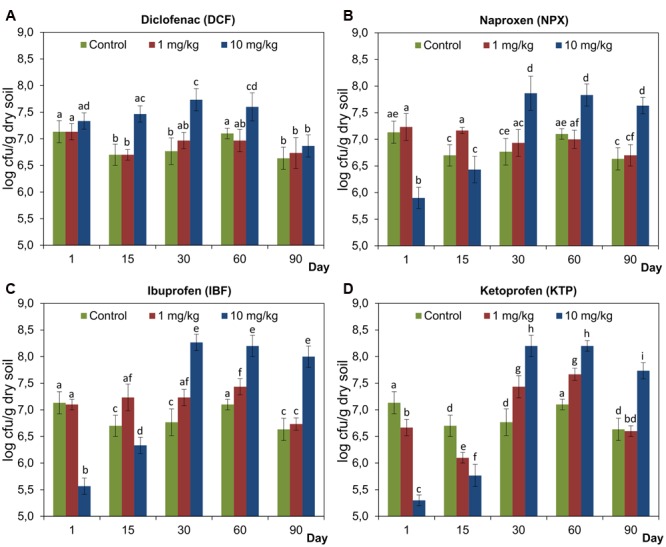
**The total number of culturable bacteria in control soil and soil treated with diclofenac (A)**, naproxen **(B)**, ibuprofen **(C)**, and ketoprofen **(D)** at concentrations of 1 and 10 mg/kg soil during the 90-day experiment. The data presented are the means and standard deviations of three replicates and are expressed as log cfu/g dry soil. Different letters (within each drug) indicate significant differences (*P* < 0.005, LSD test) related to the effects of concentration and time.

The plate-count data showed different effects of NSAIDs on the total number of culturable bacteria and this effect was more pronounced in the soil treated with higher doses of the drugs (**Figure 9**). However, the inhibitory effect of NPX, IBF, and KTP on culturable bacteria was observed on days 1 and 15 after the drug was applied. In addition, a lower dose of KTP also negatively affected the number of bacteria; however, this effect was detected only on day 1 of the experiment (**Figure 9**). At the subsequent sampling times, a stimulatory effect of higher doses of NSAIDs was observed and the number of culturable bacteria was significantly higher than those enumerated for the control soil. At lower concentrations of NSAIDs, the effects were negligible or transient (**Figure 9**). The results of a two-way ANOVA showed that the number of culturable bacteria was significantly affected by all of the factors tested in all cases. The concentration effect explained most of the variance (43%) for NPX, while the lowest variance (0.9%) was observed for KTP. The time effect had the greatest impact on KTP and it accounted for 50.2% of the variance. On the other hand, up to 68.3 and 61.4% of the observed variability accounted for the interaction between the concentration and the time for NPX and IBF, respectively. The detailed results of a two-way ANOVA analysis are shown in Supplementary Table S1. Taking into account the three factors, the ANOVA indicated that the drug and the concentration significantly affected (*P* < 0.001) the number of bacteria for which the observed variabilities were 0.9 and 5.1%, respectively, whereas incubation time, which explained 23.6% of variance, had a pronounced effect on these bacteria. In addition, significant effects (*P* < 0.001) on the number of culturable bacteria were found for all of the possible interactions between the factors tested. The highest value of variability (37.6%) was observed for the interaction between the concentration and the time. The detailed results of a three-way ANOVA analysis are shown in Supplementary Table S2.

### Number of Culturable Fungi

**FIGURE 10:**
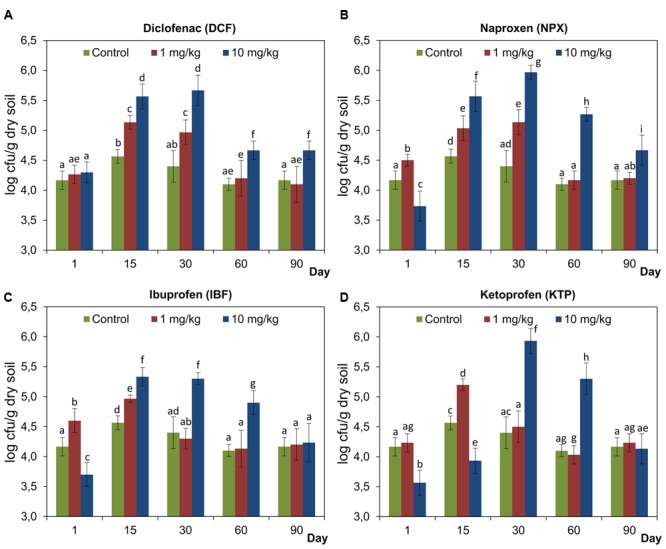
**The number of culturable fungi in control soil and soil treated with diclofenac (A)**, naproxen **(B)**, ibuprofen **(C)**, and ketoprofen **(D)** at concentrations of 1 and 10 mg/kg soil during the 90-day experiment. The data presented are the means and standard deviations of three replicates and are expressed as log cfu/g dry soil. Different letters (within each drug) indicate significant differences (*P* < 0.005, LSD test) related to the effects of concentration and time.

The enumeration of culturable fungi in the soil treated with selected NSAIDs generally showed that this group of microorganisms was significantly stimulated (**Figure 10**). At lower doses of NPX, IBF, and KTP stimulation of fungi after 15 days and 15, 30, and 60 days and after 30 and 60 days was observed, respectively. However, for the soil treated with KTP at a concentration of 1 mg/kg soil, the number of fungi was significantly lower on days 1 and 15 after the drug was applied. On the other hand, DCF used at lower dose did not affect the number of culturable fungi throughout the experimental period (**Figure 10**). Higher doses of NPX, IBF, and KTP (10 mg/kg soil), after an initial inhibition, stimulated these group of microorganisms until the end of the incubation period. By contrast, DCF did not inhibit the fungi and in this case, an increased number of fungi was observed until day 60 (**Figure 10**). The results of a two-way ANOVA showed that the number of culturable fungi was significantly affected by all of the factors tested in all cases. Among the sources of variance, the concentration effect explained most of the variance for DCF (29.5%) while the lowest value of variability was observed for KTP (3.9%). The highest contributions of the time effect and the interaction between the factors tested in the variability were found for DCF (50%) and KTP (60.2%), respectively. The detailed results of a two-way ANOVA analysis are shown in Supplementary Table S1. The ANOVA that included the three factors tested showed a significant effect of the drug (*P* < 0.001), the concentration (*P* < 0.001) and the time (*P* < 0.001) on the number of culturable fungi. When the percentage of variation was analyzed, the largest contribution was observed for the time effect (34.7%), while the drug and concentration effects explained 2.6 and 15.3% of the variance, respectively. In addition, significant effects of all the possible interactions between the factors tested on the fungal population were found, for which the highest value of variability (23.8%) was observed for the interaction between the concentration and the time. The detailed results of a three-way ANOVA analysis are shown in Supplementary Table S2.

### Principal Component Analysis

**FIGURE 11:**
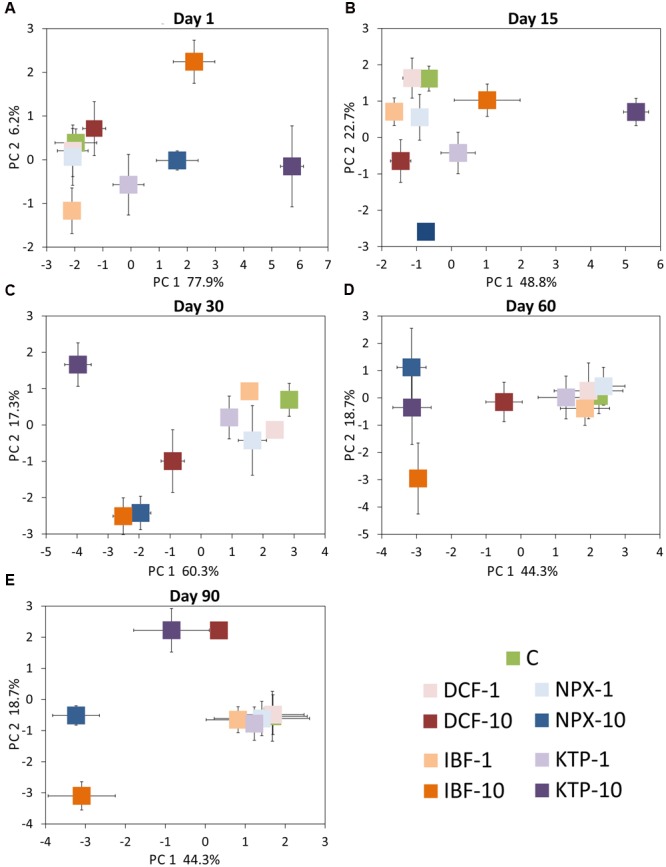
**Principal component plots generated from the measured parameters on days 1 (A)**, 15 **(B)**, 30 **(C)**, 60 **(D)**, and 90 **(E)**. C, control soil; DCF-1, soil treated with diclofenac – 1 mg/kg soil; DCF-10, soil treated with diclofenac – 10 mg/kg soil; NPX-1, soil treated with naproxen – 1 mg/kg soil; NPX-10, soil treated with naproxen – 10 mg/kg soil; IBF-1, soil treated with ibuprofen – 1 mg/kg soil; IBF-10, soil treated with ibuprofen – 10 mg/kg soil; KTP-1, soil treated with ketoprofen – 1 mg/kg soil and KTP-10, soil treated with ketoprofen – 10 mg/kg soil.

**FIGURE 12:**
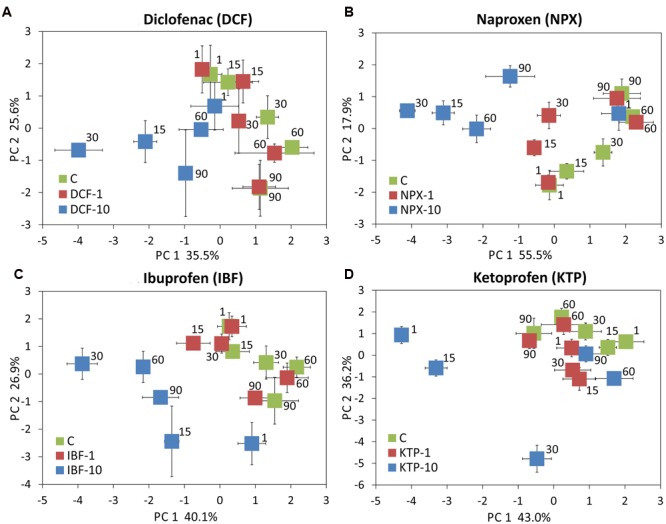
**Principal component plots generated from the measured parameters for diclofenac (A)**, naproxen **(B)**, ibuprofen **(C)**, and ketoprofen **(D)** on days 1, 15, 30, 60, and 90. C, control soil; DCF-1, soil treated with diclofenac – 1 mg/kg soil; DCF-10, soil treated with diclofenac – 10 mg/kg soil; NPX-1, soil treated with naproxen – 1 mg/kg soil; NPX-10, soil treated with naproxen – 10 mg/kg soil; IBF-1, soil treated with ibuprofen – 1 mg/kg soil; IBF-10, soil treated with ibuprofen – 10 mg/kg soil; KTP-1, soil treated with ketoprofen – 1 mg/kg soil and KTP-10, soil treated with ketoprofen – 10 mg/kg soil.

The PCA plots performed for individual sampling days allowed a snapshot of the observed variation in the total microbial activity for all of the drugs and dosages tested at individual incubation points to be assessed (**Figure 11**). In general, a significant impact of the drug concentration, rather than the incubation time was noted because on the PCA scatterplots samples separated in the form of a gradient that overlapped the tested concentrations; however, the most distinct separation was observed for the KTP samples, especially on days 1, 15, and 30 (**Figure 11**). However, a MANOVA analysis revealed that 56.4, 89.7, 87.2, and 64.9% of the total variation in PC1 was dose-related for days 1, 30, 60, and 90, respectively. The variation turned out to be affected more by the type of drug and interaction between the type of drug and the concentration only on day 15. In PC2, the variation was predominantly affected by the concentration on days 1 and 15 (24.7 and 59.9%, respectively), while the impact of the type of drug or the interaction between the drug and the concentration was predominant on days 60 and 90. The detailed results of a MANOVA analysis are shown in Supplementary Table S3. PCA performed for the individual drugs enabled the scattering of the samples with regard to the incubation time and concentrations tested (**Figure 12**). Similar to the previous analyses, the PCA plots revealed a predominant effect of the concentration, 49.8, 42.2, 50.9% for DCF, NPX, and IBF in PC1, respectively, whereas the PC1 scores for KTP varied more with regard to the interaction of the concentration and the incubation time. Similarly, the variations obtained for PC2 were predominantly affected by the incubation time only for the DCF and NPX samples – 65.1 and 45.3% of their total variation, respectively. The detailed results of a MANOVA analysis are shown in Supplementary Table S4.

## Discussion

In this study we evaluated the microbial response to selected commercially available NSAIDs by investigating some biochemical processes in loamy sand soil. Currently, there are no reports related to the effects of NSAIDs on the soil microbial activity, and therefore it is difficult to refer to results obtained by other authors. In general, the obtained data showed a stimulatory effect of NSAIDs on the microbial activity. Higher concentrations of the drugs caused a greater effect, which was observed during the entire incubation period. However, an inhibitory effect of some NSAIDs was observed at the beginning of the experiment. At lower concentrations of the drugs, the effects were negligible or transient. Since commercially available NSAIDs contain some additional organic ingredients (e.g., lactose, sucrose, starch, gelatin, etc.), the input of these compounds into soil could stimulate biochemical processes in the soil as a result of the use them by soil microorganism as additional sources of carbon and energy.

The obtained results might not only be related to the individual features of a microbial population but also to the type of soil. The soil used in this study was characterized by a low content of organic matter and a high content of the sand fraction, which might result in a lower adsorption of NSAIDs to soil components and a higher availability of drugs for soil microorganisms (Xu et al., 2010; González-Naranjo et al., 2013; Vasiliadou et al., 2013; Estevez et al., 2014; Graouer-Bacart et al., 2016). This effect was also observed in relation to other organic compounds such as pesticides (Karpouzas and Walker, 2000; Liang et al., 2011; Cycoń et al., 2014), polycyclic aromatic hydrocarbons (Sverdrup et al., 2002; Rivas et al., 2008; Chen and Yuan, 2011) and other pharmaceuticals (Topp et al., 2008b; Sabourin et al., 2009; Al-Rajab et al., 2015; Barra Caracciolo et al., 2015). In addition, the observed effect might also be related to the chemical structure and physico-chemical properties of an individual NSAID that determine its toxicity to soil microorganisms (Dastidar et al., 2000; Paje et al., 2002; Lawrence et al., 2007) and the susceptibility to degradation processes (Xu et al., 2009; Al-Rajab et al., 2010; Lin and Gan, 2011; Grossberger et al., 2014; Domaradzka et al., 2015). Since NSAIDs belong to the compounds that are characterized by hydrophobic properties, their action in soil is highly dependent on their solubility in water and hence on the availability of these drugs for microbial cells. Due to the low solubility of many NSAIDs, their availability for microorganisms may be limited and therefore their effects would be negligible, transient or unnoticeable. On the other hand, a low solubility may contribute to the long-term persistence of the chemicals in the soil environment (Karpouzas and Walker, 2000; Xu et al., 2009; Cycoń et al., 2014; Al-Rajab et al., 2015). In general, in our study we observed the weakest effect for DCF, which among the NSAIDs tested, is characterized by the lowest solubility in water at a level of 2.4 mg/L, while the greatest effect was observed for KTP, whose solubility is 21-fold greater (51 mg/L) than DCF. Moreover, Xu et al. (2009) observed that KTP was not strongly adsorbed to soil particles, which suggests that it has a high potential to move downward with percolating water and contaminate the ground water. However, a significant effect on the measured microbial activities in the soil was also found for NPX and IBF.

The results obtained from the analysis of the respiratory activity of the soil indicated that DCF and NPX did not inhibit this activity and even stimulated it. The observed effect might be related to the capability of some microorganisms to use these drugs as sources of carbon and energy for growth, thus resulting in an increase in soil respiration (Marco-Urrea et al., 2010c; Rodarte-Morales et al., 2012; Almeida et al., 2013; Murdoch and Hay, 2015). On the other hand, there are some NSAIDs (e.g., naproxen, diclofenac) that are not directly utilizable by soil microorganisms and these may negatively affect the metabolic activity and integrity of microbial cells, thereby causing a decrease in soil respiration activity (Elvers and Wright, 1995; Dastidar et al., 2000; Wojcieszyńska et al., 2014). This effect was observed for IBF and KTP at the beginning of the experiment; however, the stimulation of this activity was found at the subsequent sampling times. Since the chemical structure and physico-chemical properties of the organic compounds determine their toxicity to microorganisms, their effects on soil respiration were found to be extremely diversified (Liu et al., 2009; Kotzerke et al., 2011; Conkle and White, 2012; Cycoń and Piotrowska-Seget, 2015; Álvarez-Martín et al., 2016).

Assessment of enzyme activities is considered to be a valuable tool that can indicate the potential of soil to support the biochemical processes that are essential to maintain soil fertility. Any NSAID applications that influence microbial communities and their biochemical activities in soil may be expected to generate changes in the soil enzyme activity level (Gianfreda et al., 1994; Alef, 1995; Gil-Sotres et al., 2005). When analyzing the activity of DHA in soil treated with selected NSAIDs, we found that higher doses of all of the drugs negatively affected this activity. The largest decrease was recorded when KTP was applied. However, in this and other cases, the adverse effects were observed up to day 30 of the experiment. Dehydrogenases occur intracellularly in all living microbial cells and they are linked to the microbial respiration processes and reflect the physiological state of microorganisms (Alef, 1995; Marx et al., 2005). Surprisingly, in our study the activity of DHA was inhibited at the beginning of the experiment when NSAIDs were applied, whereas at the same time the soil respiration was higher for the soils treated with NSAIDs. This phenomenon may have resulted from the fact that the level of TTC reduction was used to estimate the DHA activity. Since dehydrogenases are a broad group of enzymes responsible for the transfer of hydrogen from various organic substrates to an electron acceptor, the process of TTC reduction by specific DHA may be sensitive to the NSAIDs used. Moreover, a low activity of DHA in soil treated with NSAIDs might be associated with the death of the drug-sensitive part of the microbial population. Similar effects have been demonstrated by many authors in relation to some organic compounds including other pharmaceuticals and pesticides (Fließbach and Mäder, 2004; Cycoń et al., 2010; Conkle and White, 2012; Cui et al., 2013). Dehydrogenases released from dead cells do not accumulate in soil since they undergo rapid degradation. The released fraction of enzymes is unstable, has a short-lived activity and is subject to rapid denaturation, degradation or is irreversibly inhibited (Marx et al., 2005). In contrast, as is shown by the data obtained in this study, the activities of PHOS-H, PHOS-OH and URE were generally stimulated by the NSAIDs used; however, a short-term inhibition was observed for the activities of both phosphatases in soil treated with higher doses of NPX, IBF and KTP. In contrast to DHA, which are extracellular enzymes, PHOSs and URE are immobilized by the organic and non-organic soil colloids that protect them from degradation (Boyd and Mortland, 1990). A certain proportion of free enzymes may be stabilized through adsorption into humic materials, which despite affecting their catalytic potential may maintain the enzyme activity in the soil (Badiane et al., 2001). Moreover, an increase in the activity of phosphatases in soil treated with NSAIDs may have been associated with the stimulation of the indigenous fungi whose number significantly increased over the experimental period, and these microorganisms are known as soil producers of phosphatases (Nannipieri et al., 1990) and NSAID-degrading microorganisms (Hata et al., 2010; Marco-Urrea et al., 2010c; Rodarte-Morales et al., 2012).

In the risk assessment of organic contaminants toward microbial-mediated processes, their impact on nitrification and ammonification rates are commonly used. It is well-known that the nitrification process is more sensitive to various chemicals than the ammonification process. The first is carried out by a small group of microorganisms, while the second is mediated by a wide range of soil microorganisms (Hicks et al., 1990). The results of our study showed that the nitrification and ammonification rates were generally stimulated by the NSAIDs used as was indicated by the increased concentrations of N-NO_3_^-^ and N-NH_4_^+^; however, an inhibitory effect of NPX and KTP on nitrification was observed at the beginning of the experiment. Although, a small group of Gram-negative microorganisms is responsible for the nitrification in soil, they may be insensitive to the NSAIDs used compared to Gram-positive microorganisms. Some studies have reported that NSAIDs have significant antibacterial activity against Gram-positive bacteria (Elvers and Wright, 1995; Dastidar et al., 2000). In addition, the increase in the concentration of N-NO_3_^-^ in the soils treated with NSAIDs might result from the stimulation of the ammonification process, as was indicated by the increased concentration of N-NH_4_^+^, which is the substrate for the nitrifiers (Hicks et al., 1990). The low sensitivity of nitrifiers to NSAIDs was proven by Kraigher and Mandic-Mulec (2011), who observed that the addition of different concentrations (0, 0.05, 0.2, 0.5 mg/L) of IBF, NPX, KTP, and DCF did not affect ammonia removal. However, the eﬄuent concentration of N-NO_2_^-^ and N-NO_3_^-^ was significantly higher in the two reactors that were operated with 0.05 mg/L of NSAIDs than in the other reactors. The observed stimulation of ammonification in the NSAID-treated soils can be explained by the fact that sensitive to drugs bacteria and Achaea responsible for oxidizing ammonia may be killed, and therefore N-NH_4_^+^ will not be transformed into either nitrite or various nitrogen oxides in the soils (Kinney et al., 2005). Another reason for this might be related to the death of some of the microorganisms that are then subsequently mineralized and consequently lead to increased concentrations of ammonium. It is reasonable to assume that some components of the NSAID formulations could stimulate ammonifying bacteria, thus resulting in the enhanced production of ammonium as well.

As was shown by the plate-count data, the NSAIDs negatively affected the number of culturable bacteria and fungi at the beginning of the experiment. However, a stimulation of growth in both groups of microorganisms for higher doses of NSAIDs was found at the subsequent sampling times. Conversely, some studies have reported that NPX, IBF, and KTP had significant antibacterial and antifungal activity (Sanyal et al., 1993; Elvers and Wright, 1995; Dastidar et al., 2000). For example, Paje et al. (2002) reported that lotic biofilms composed of bacterial and algal populations lost about 70% of their overall initial biomass following 4 weeks of exposure to 0.1 mg/L DCF. However, some *Cytophaga* bacteria survived and were able to degrade up to 97% of the parent compound within 5 days of its application, thus demonstrating the capability of some microorganisms to adapt to this compound. In another work of Lawrence et al. (2007), river biofilms were exposed to environmentally relevant (0.01–0.1 mg/L) DCF concentrations over two seasons. At the lower concentration in spring, there was no significant effect on algal, bacterial and cyanobacterial biomass, whereas in the summer, the biomass of cyanobacteria was reduced. In contrast, at the highest concentration in spring, the cyanobacterial biomass and biofilm thickness increased. The use of fluorescence *in situ* hybridization (FISH) and DGGE methods allowed them to find DCF-induced differences in the community structures. In addition to the changes in bacterial community, this drug also caused changes in the profiles of carbon utilization as measured by Biolog method. Lawrence et al. (2005) also found that IBF reduced the overall bacterial biomass of a riverine biofilm community originated from rotating annular bioreactors and exposed for 8 weeks to a concentration of 0.01 mg/L. Moreover, a FISH analysis revealed changes in the composition of the microbial biofilm exposed to IBF during the experiment. In particular, *Cyanobacteria* together with Gamma-*Proteobacteria* and Gram-positive bacteria *Firmicutes* decreased significantly, while Alpha-, Beta-*Proteobacteria, Cytophaga–Flavobacteria* and sulfate-reducing bacteria increased, thus suggesting a role of these groups in IBF biodegradation.

The increase in the numbers of culturable bacteria and fungi that was observed in our study may have resulted from the adaptation of the bacteria and fungi to the NSAIDs applied. Many soil microorganisms have evolved the ability to degrade NSAIDs and to use them as an additional source of carbon and energy for their growth. This may be proven by the lack of a lag phase and immediate start of mineralization that was observed during studies on the biodegradation of IBF (20 mg/kg) in soil (Girardi et al., 2013). Moreover, the ability of some microorganisms to grow in the presence of NSAIDs may result from the NSAID-induced death of other microorganisms (Dastidar et al., 2000; Paje et al., 2002; Lawrence et al., 2007). Microorganisms that are not sensitive to NSAIDs may utilize the nutrients that are released from dead cells, thus resulting in an increase in their numbers. It has been reported that the abiotic degradation of NSAIDs plays a role in many cases; however, this process is slow under anaerobic or sterile conditions. Many studies have indicated the key role of microorganisms in the transformation of DCF, NPX, IBF, KTP, and other pharmaceuticals in soils that have different characteristics (Xu et al., 2009; Carr et al., 2011a,b; Girardi et al., 2013; Nowak et al., 2013; Al-Rajab et al., 2015).

## Conclusion

Non-steroidal anti-inflammatory drugs enter the aquatic and terrestrial systems continuously and it is not possible to predict their environmental concentration and their impact on a wide range of living biota in the future. To date studies concerning the impact of NSAIDs on organisms have mainly been conducted in aquatic systems, and therefore little is known about the potential effects of non-inflammatory drugs on non-target organisms such as soil microorganism. Only a few studies have been carried out on the effect of some NSAIDs on the microbial community structure and carbon-source utilizing profiles. In terms of the microbial community, the reduction or loss of catabolic properties is an undesirable effect and may disturb the functioning of soil. Our study presents evidence that the application of DCF, NPX, IBF, and KTP altered the number and activity of soil microorganisms. However, it is not possible to unambiguously assess the effect of NSAIDs on soil microorganisms because a drug’s fate, dissipation rate, and antimicrobial activity are determined by a combination of compound-specific features as well as many environmental conditions. Therefore, more studies should be performed in order to gain more detailed knowledge on mode of action of NSAIDs on microorganisms and the environmental properties that affect the sorption, persistence, transport, accumulation, and degradation of NSAIDs in soil. Moreover, it appears that there is an urgent need to monitor the occurrence of these chemicals in the eﬄuents from wastewater treatment plants, sewage sludge and water that is used for field irrigation. Since NSAIDs are thought to be emerging contaminants, it is necessary to determine the ecotoxicological risk that results from their presence in soils.

## Author Contributions

Conceived and designed experiments: MC. Contributed reagents and performed experiments: MC and BŻ. Prepared the figures and tables: MC and SB. Analyzed results: MC, SB, and ZP-S. Wrote the paper: MC and ZP-S.

## Conflict of Interest Statement

The authors declare that the research was conducted in the absence of any commercial or financial relationships that could be construed as a potential conflict of interest.
