# Supplementary material for: Variable Effects of Non-steroidal Anti-inflammatory Drugs (NSAIDs) on Selected Biochemical Processes Mediated by Soil Microorganisms
Source: Front Microbiol. 2016 Dec 5;7:1969. doi: 10.3389/fmicb.2016.01969 (PMC5147054; doi:10.3389/fmicb.2016.01969)
Supplement: Supplementary file 1 [file Table_1.DOC]

**TABLE S1 Results of a two-way ANOVA for the effects of concentration of drug, time and their interaction on measured biochemical and microbial parameters.**

| **Parameter** | **Drug** | **Source of variation** | ***df*** | **Sum of squares** | **Mean squares** | ***F*** | ***P*** | **Variance explained (%)** |
| --- | --- | --- | --- | --- | --- | --- | --- | --- |
| SIR | DCF | Concentration (C) | 2 | 46.0 | 23.0 | 18.5 | ***P* < 0.001** | 30.4 |
|  |  | Time (T) | 4 | 29.9 | 7.5 | 6.0 | ***P* = 0.001** | 19.8 |
|  |  | C × T | 8 | 37.9 | 4.7 | 3.8 | ***P* = 0.003** | 25.1 |
|  | NPX | Concentration (C) | 2 | 24.8 | 12.4 | 20.7 | ***P* < 0.001** | 19.2 |
|  |  | Time (T) | 4 | 28.4 | 7.1 | 11.8 | ***P* < 0.001** | 21.9 |
|  |  | C × T | 8 | 58.0 | 7.3 | 12.1 | ***P* < 0.001** | 44.9 |
|  | IBF | Concentration (C) | 2 | 19.7 | 9.9 | 8.7 | ***P* = 0.001** | 7.2 |
|  |  | Time (T) | 4 | 70.1 | 17.5 | 15.5 | ***P* < 0.001** | 25.7 |
|  |  | C × T | 8 | 148.9 | 18.6 | 16.4 | ***P* < 0.001** | 54.6 |
|  | KTP | Concentration (C) | 2 | 64.1 | 32.1 | 15.7 | ***P* < 0.001** | 9.0 |
|  |  | Time (T) | 4 | 217.9 | 54.5 | 26.7 | ***P* < 0.001** | 30.5 |
|  |  | C × T | 8 | 371.4 | 46.4 | 22.7 | ***P* < 0.001** | 52.0 |
| DHA | DCF | Concentration (C) | 2 | 10.9 | 5.5 | 2.4 | *P* = 0.106 | 6.2 |
|  |  | Time (T) | 4 | 80.4 | 20.1 | 8.9 | ***P* < 0.001** | 45.3 |
|  |  | C × T | 8 | 17.9 | 2.2 | 1.0 | *P* = 0.465 | 10.1 |
|  | NPX | Concentration (C) | 2 | 0.2 | 0.1 | 0.03 | *P* = 0.969 | 0.1 |
|  |  | Time (T) | 4 | 64.4 | 16.1 | 5.8 | ***P* = 0.001** | 34.3 |
|  |  | C × T | 8 | 39.8 | 4.9 | 1.8 | *P* = 0.118 | 21.2 |
|  | IBF | Concentration (C) | 2 | 47.6 | 23.8 | 11.8 | ***P* < 0.001** | 16.6 |
|  |  | Time (T) | 4 | 114.6 | 28.6 | 14.2 | ***P* < 0.001** | 39.9 |
|  |  | C × T | 8 | 64.4 | 8.0 | 3.9 | ***P* = 0.002** | 22.4 |
|  | KTP | Concentration (C) | 2 | 102.5 | 51.3 | 18.0 | ***P* < 0.001** | 32.2 |
|  |  | Time (T) | 4 | 33.2 | 8.3 | 2.9 | ***P* < 0.037** | 10.4 |
|  |  | C × T | 8 | 97.7 | 12.2 | 4.3 | ***P* = 0.002** | 30.6 |
| PHOS-H | DCF | Concentration (C) | 2 | 1152.2 | 576.1 | 77.2 | ***P* < 0.001** | 54.2 |
|  |  | Time (T) | 4 | 344.5 | 86.1 | 11.5 | ***P* < 0.001** | 16.2 |
|  |  | C × T | 8 | 406.3 | 50.8 | 6.8 | ***P* < 0.001** | 19.1 |
|  | NPX | Concentration (C) | 2 | 321.1 | 160.6 | 21.0 | ***P* < 0.001** | 14.5 |
|  |  | Time (T) | 4 | 664.6 | 166.1 | 21.7 | ***P* < 0.001** | 30.0 |
|  |  | C × T | 8 | 999.6 | 124.9 | 16.3 | ***P* < 0.001** | 45.1 |
|  | IBF | Concentration (C) | 2 | 218.8 | 109.4 | 15.4 | ***P* < 0.001** | 14.5 |
|  |  | Time (T) | 4 | 607.8 | 151.9 | 21.4 | ***P* < 0.001** | 40.2 |
|  |  | C × T | 8 | 471.2 | 58.9 | 8.3 | ***P* < 0.001** | 31.2 |
|  | KTP | Concentration (C) | 2 | 22.0 | 11.0 | 1.6 | *P* = 0.212 | 0.7 |
|  |  | Time (T) | 4 | 716.1 | 179.0 | 26.5 | ***P* < 0.001** | 24.1 |
|  |  | C × T | 8 | 2028.4 | 253.6 | 37.5 | ***P* < 0.001** | 68.3 |
| PHOS-OH | DCF | Concentration (C) | 2 | 98.7 | 49.4 | 17.4 | ***P* < 0.001** | 16.6 |
|  |  | Time (T) | 4 | 314.1 | 78.5 | 27.6 | ***P* < 0.001** | 52.7 |
|  |  | C × T | 8 | 97.5 | 12.2 | 4.3 | ***P* = 0.002** | 16.4 |
|  | NPX | Concentration (C) | 2 | 321.2 | 160.6 | 38.7 | ***P* < 0.001** | 29.8 |
|  |  | Time (T) | 4 | 161.6 | 40.4 | 9.7 | ***P* < 0.001** | 15.0 |
|  |  | C × T | 8 | 470.8 | 58.8 | 14.2 | ***P* < 0.001** | 43.7 |
|  | IBF | Concentration (C) | 2 | 89.7 | 44.9 | 11.2 | ***P* < 0.001** | 7.1 |
|  |  | Time (T) | 4 | 75.3 | 18.8 | 4.7 | ***P* = 0.005** | 6.0 |
|  |  | C × T | 8 | 978.9 | 122.4 | 30.6 | ***P* < 0.001** | 77.4 |
|  | KTP | Concentration (C) | 2 | 40.1 | 20.1 | 4.5 | ***P* = 0.019** | 2.4 |
|  |  | Time (T) | 4 | 256.3 | 64.1 | 14.4 | ***P* < 0.001** | 15.3 |
|  |  | C × T | 8 | 1243.4 | 155.4 | 35.0 | ***P* < 0.001** | 74.3 |

*DCF, diclofenac; NPX, naproxen; IBF, ibuprofen; KTP, ketoprofen. The effects in bold are significant at P < 0.05.*

**TABLE S1 Continued**

| **Parameter** | **Drug** | **Source of variation** | ***df*** | **Sum of squares** | **Mean squares** | ***F*** | ***P*** | **Variance explained (%)** |
| --- | --- | --- | --- | --- | --- | --- | --- | --- |
| URE | DCF | Concentration (C) | 2 | 69.6 | 34.8 | 26.6 | ***P* < 0.001** | 34.5 |
|  |  | Time (T) | 4 | 46.4 | 11.6 | 8.9 | ***P* < 0.001** | 23.0 |
|  |  | C × T | 8 | 46.4 | 5.8 | 4.4 | ***P* = 0.001** | 23.0 |
|  | NPX | Concentration (C) | 2 | 284.6 | 142.3 | 90.2 | ***P* < 0.001** | 62.0 |
|  |  | Time (T) | 4 | 72.1 | 18.0 | 11.4 | ***P* < 0.001** | 15.7 |
|  |  | C × T | 8 | 55.2 | 6.9 | 4.4 | ***P* = 0.001** | 12.0 |
|  | IBF | Concentration (C) | 2 | 123.5 | 61.8 | 54.5 | ***P* < 0.001** | 57.9 |
|  |  | Time (T) | 4 | 41.5 | 10.4 | 9.2 | ***P* < 0.001** | 19.4 |
|  |  | C × T | 8 | 14.3 | 1.8 | 1.6 | *P* = 0.175 | 6.7 |
|  | KTP | Concentration (C) | 2 | 182.9 | 91.5 | 76.2 | ***P* < 0.001** | 49.2 |
|  |  | Time (T) | 4 | 83.8 | 20.9 | 17.5 | ***P* < 0.001** | 22.5 |
|  |  | C × T | 8 | 69.3 | 8.7 | 7.2 | ***P* < 0.001** | 18.6 |
| N-NO_3_^-^ | DCF | Concentration (C) | 2 | 0.2 | 0.1 | 0.2 | *P* = 0.799 | 0.3 |
|  |  | Time (T) | 4 | 47.1 | 11.8 | 29.9 | ***P* < 0.001** | 75.3 |
|  |  | C × T | 8 | 3.5 | 0.4 | 1.1 | *P* = 0.387 | 5.6 |
|  | NPX | Concentration (C) | 2 | 4.3 | 2.1 | 7.7 | ***P* = 0.002** | 7.9 |
|  |  | Time (T) | 4 | 27.4 | 6.9 | 24.6 | ***P* < 0.001** | 50.4 |
|  |  | C × T | 8 | 14.3 | 1.8 | 6.4 | ***P* < 0.001** | 26.3 |
|  | IBF | Concentration (C) | 2 | 26.6 | 13.3 | 33.6 | ***P* < 0.001** | 28.7 |
|  |  | Time (T) | 4 | 42.4 | 10.6 | 26.8 | ***P* < 0.001** | 45.8 |
|  |  | C × T | 8 | 11.8 | 1.5 | 3.7 | ***P* = 0.004** | 12.7 |
|  | KTP | Concentration (C) | 2 | 0.6 | 0.3 | 0.8 | *P* = 0.473 | 0.6 |
|  |  | Time (T) | 4 | 34.1 | 8.5 | 20.4 | ***P* < 0.001** | 33.0 |
|  |  | C × T | 8 | 56.3 | 7.0 | 16.8 | ***P* < 0.001** | 54.3 |
| N-NH_4_^+^ | DCF | Concentration (C) | 2 | 24.8 | 12.4 | 8.3 | ***P* = 0.001** | 17.6 |
|  |  | Time (T) | 4 | 21.1 | 5.3 | 3.5 | ***P* = 0.018** | 15.0 |
|  |  | C × T | 8 | 50.5 | 6.3 | 4.2 | ***P* = 0.002** | 35.8 |
|  | NPX | Concentration (C) | 2 | 339.6 | 169.8 | 114.0 | ***P* < 0.001** | 58.6 |
|  |  | Time (T) | 4 | 112.1 | 28.0 | 18.8 | ***P* < 0.001** | 19.4 |
|  |  | C × T | 8 | 82.8 | 10.4 | 6.9 | ***P* < 0.001** | 14.3 |
|  | IBF | Concentration (C) | 2 | 478.0 | 239.0 | 195.6 | ***P* < 0.001** | 69.5 |
|  |  | Time (T) | 4 | 60.3 | 15.1 | 12.3 | ***P* < 0.001** | 8.8 |
|  |  | C × T | 8 | 112.6 | 14.1 | 11.5 | ***P* < 0.001** | 16.4 |
|  | KTP | Concentration (C) | 2 | 435.9 | 217.9 | 144.2 | ***P* < 0.001** | 60.1 |
|  |  | Time (T) | 4 | 86.0 | 21.5 | 14.2 | ***P* < 0.001** | 11.8 |
|  |  | C × T | 8 | 158.5 | 19.8 | 13.1 | ***P* < 0.001** | 21.8 |
| Bacteria | DCF | Concentration (C) | 2 | 2.7 | 1.3 | 34.2 | ***P* < 0.001** | 43.0 |
|  |  | Time (T) | 4 | 1.5 | 0.4 | 9.5 | ***P* < 0.001** | 23.9 |
|  |  | C × T | 8 | 0.9 | 0.1 | 2.9 | ***P* = 0.018** | 14.3 |
|  | NPX | Concentration (C) | 2 | 0.5 | 0.3 | 5.9 | ***P* = 0.007** | 4.1 |
|  |  | Time (T) | 4 | 2.2 | 0.6 | 12.4 | ***P* < 0.001** | 17.2 |
|  |  | C × T | 8 | 8.8 | 1.1 | 24.6 | ***P* < 0.001** | 68.3 |
|  | IBF | Concentration (C) | 2 | 1.3 | 0.7 | 20.2 | ***P* < 0.001** | 5.8 |
|  |  | Time (T) | 4 | 6.3 | 1.6 | 48.9 | ***P* < 0.001** | 28.4 |
|  |  | C × T | 8 | 13.7 | 1.7 | 53.0 | ***P* < 0.001** | 61.4 |
|  | KTP | Concentration (C) | 2 | 0.3 | 0.1 | 4.6 | ***P* = 0.018** | 0.9 |
|  |  | Time (T) | 4 | 15.2 | 3.8 | 133.2 | ***P* < 0.001** | 50.2 |
|  |  | C × T | 8 | 13.9 | 1.7 | 61.2 | ***P* < 0.001** | 46.1 |

*DCF, diclofenac; NPX, naproxen; IBF, ibuprofen; KTP, ketoprofen. The effects in bold are significant at P < 0.05.*

**TABLE S1 Continued**

| **Parameter** | **Drug** | **Source of variation** | ***df*** | **Sum of squares** | **Mean squares** | ***F*** | ***P*** | **Variance explained (%)** |
| --- | --- | --- | --- | --- | --- | --- | --- | --- |
| Fungi | DCF | Concentration (C) | 2 | 3.7 | 1.8 | 47.5 | ***P* < 0.001** | 29.5 |
|  |  | Time (T) | 4 | 6.3 | 1.6 | 40.2 | ***P* < 0.001** | 50.0 |
|  |  | C × T | 8 | 1.4 | 0.2 | 4.5 | ***P* = 0.001** | 11.1 |
|  | NPX | Concentration (C) | 2 | 5.1 | 2.6 | 70.6 | ***P* < 0.001** | 25.7 |
|  |  | Time (T) | 4 | 8.2 | 2.1 | 56.8 | ***P* < 0.001** | 41.4 |
|  |  | C × T | 8 | 5.4 | 0.7 | 18.8 | ***P* < 0.001** | 27.4 |
|  | IBF | Concentration (C) | 2 | 1.3 | 0.7 | 16.5 | ***P* < 0.001** | 12.5 |
|  |  | Time (T) | 4 | 4.1 | 1.0 | 25.9 | ***P* < 0.001** | 39.2 |
|  |  | C × T | 8 | 3.9 | 0.5 | 12.2 | ***P* < 0.001** | 36.9 |
|  | KTP | Concentration (C) | 2 | 0.6 | 0.3 | 8.8 | ***P* = 0.001** | 3.9 |
|  |  | Time (T) | 4 | 4.9 | 1.2 | 33.2 | ***P* < 0.001** | 29.3 |
|  |  | C × T | 8 | 10.0 | 1.3 | 34.0 | ***P* < 0.001** | 60.2 |

*DCF, diclofenac; NPX, naproxen; IBF, ibuprofen; KTP, ketoprofen. The effects in bold are significant at P < 0.05.*
