# Supplementary material for: Variable Effects of Non-steroidal Anti-inflammatory Drugs (NSAIDs) on Selected Biochemical Processes Mediated by Soil Microorganisms
Source: Front Microbiol. 2016 Dec 5;7:1969. doi: 10.3389/fmicb.2016.01969 (PMC5147054; doi:10.3389/fmicb.2016.01969)
Supplement: Supplementary file 2 [file Table_2.DOC]

**TABLE S2 Results of a three-way ANOVA for the effects of drug, concentration, time and their interaction on measured biochemical and microbial parameters.**

| **Parameter** | **Source of variation** | ***df*** | **Sum of squares** | **Mean squares** | ***F*** | ***P*** | **Variance explained (%)** |
| --- | --- | --- | --- | --- | --- | --- | --- |
| SIR | Drug (D) | 3 | 140.2 | 46.7 | 42.7 | ***P* < 0.001** | 10.1 |
|  | Concentration (C) | 2 | 2.4 | 1.2 | 1.1 | *P* = 0.335 | 0.2 |
|  | Time (T) | 4 | 184.6 | 46.1 | 42.2 | ***P* < 0.001** | 13.3 |
|  | D × C | 6 | 131.1 | 21.9 | 20.0 | ***P* < 0.001** | 9.5 |
|  | D × T | 12 | 160.2 | 13.3 | 12.2 | ***P* < 0.001** | 11.6 |
|  | C × T | 8 | 396.1 | 49.5 | 45.2 | ***P* < 0.001** | 28.6 |
|  | D × C × T | 24 | 240.4 | 10.0 | 9.2 | ***P* < 0.001** | 17.3 |
| DHA | Drug (D) | 3 | 33.9 | 11.3 | 4.6 | ***P* = 0.004** | 3.4 |
|  | Concentration (C) | 2 | 104.5 | 52.2 | 21.1 | ***P* < 0.001** | 10.4 |
|  | Time (T) | 4 | 257.3 | 64.3 | 25.9 | ***P* < 0.001** | 25.6 |
|  | D × C | 6 | 56.9 | 9.5 | 3.8 | ***P* = 0.001** | 5.7 |
|  | D × T | 12 | 35.3 | 2.9 | 1.2 | *P* = 0.299 | 3.5 |
|  | C × T | 8 | 159.5 | 19.9 | 8.1 | ***P* < 0.001** | 15.9 |
|  | D × C × T | 24 | 60.3 | 2.5 | 1.0 | *P* = 0.455 | 6.0 |
| PHOS-H | Drug (D) | 3 | 245 | 82 | 11.3 | ***P* < 0.001** | 2.7 |
|  | Concentration (C) | 2 | 1257 | 629 | 86.8 | ***P* < 0.001** | 13.9 |
|  | Time (T) | 4 | 1486 | 371 | 51.3 | ***P* < 0.001** | 16.4 |
|  | D × C | 6 | 457 | 76 | 10.5 | ***P* < 0.001** | 5.0 |
|  | D × T | 12 | 847 | 71 | 9.8 | ***P* < 0.001** | 9.3 |
|  | C × T | 8 | 2574 | 322 | 44.4 | ***P* < 0.001** | 28.4 |
|  | D × C × T | 24 | 1331 | 55 | 7.7 | ***P* < 0.001** | 14.7 |
| PHOS-OH | Drug (D) | 3 | 195 | 65 | 16.9 | ***P* < 0.001** | 4.1 |
|  | Concentration (C) | 2 | 209 | 105 | 27.1 | ***P* < 0.001** | 4.4 |
|  | Time (T) | 4 | 366 | 92 | 23.7 | ***P* < 0.001** | 7.6 |
|  | D × C | 6 | 340 | 57 | 14.7 | ***P* < 0.001** | 7.1 |
|  | D × T | 12 | 441 | 37 | 9.5 | ***P* < 0.001** | 9.2 |
|  | C × T | 8 | 2170 | 271 | 70.2 | ***P* < 0.001** | 45.1 |
|  | D × C × T | 24 | 621 | 26 | 6.7 | ***P* < 0.001** | 12.9 |
| URE | Drug (D) | 3 | 79.4 | 26.5 | 20.3 | ***P* < 0.001** | 6.0 |
|  | Concentration (C) | 2 | 600.7 | 300.3 | 230.0 | ***P* < 0.001** | 45.3 |
|  | Time (T) | 4 | 187.2 | 46.8 | 35.9 | ***P* < 0.001** | 14.1 |
|  | D × C | 6 | 60.0 | 10.0 | 7.7 | ***P* < 0.001** | 4.5 |
|  | D × T | 12 | 56.6 | 4.7 | 3.6 | ***P* < 0.001** | 4.3 |
|  | C × T | 8 | 115.5 | 14.4 | 11.1 | ***P* < 0.001** | 8.7 |
|  | D × C × T | 24 | 69.6 | 2.9 | 2.2 | ***P* = 0.002** | 5.3 |
| N-NO_3_^-^ | Drug (D) | 3 | 10.6 | 3.5 | 9.6 | ***P* < 0.001** | 3.3 |
|  | Concentration (C) | 2 | 15.8 | 7.9 | 21.2 | ***P* < 0.001** | 4.9 |
|  | Time (T) | 4 | 130.2 | 32.6 | 87.7 | ***P* < 0.001** | 40.2 |
|  | D × C | 6 | 15.9 | 2.7 | 7.2 | ***P* < 0.001** | 4.9 |
|  | D × T | 12 | 20.9 | 1.7 | 4.7 | ***P* < 0.001** | 6.4 |
|  | C × T | 8 | 50.0 | 6.3 | 16.8 | ***P* < 0.001** | 15.4 |
|  | D × C × T | 24 | 35.8 | 1.5 | 4.0 | ***P* < 0.001** | 11.1 |
| N-NH_4_^+^ | Drug (D) | 3 | 184.0 | 61.3 | 42.9 | ***P* < 0.001** | 7.9 |
|  | Concentration (C) | 2 | 1086.0 | 543.0 | 380.3 | ***P* < 0.001** | 46.9 |
|  | Time (T) | 4 | 132.7 | 33.2 | 23.2 | ***P* < 0.001** | 5.7 |
|  | D × C | 6 | 192.3 | 32.1 | 22.5 | ***P* < 0.001** | 8.3 |
|  | D × T | 12 | 146.8 | 12.2 | 8.6 | ***P* < 0.001** | 6.3 |
|  | C × T | 8 | 245.7 | 30.7 | 21.5 | ***P* < 0.001** | 10.6 |
|  | D × C × T | 24 | 158.7 | 6.6 | 4.6 | ***P* < 0.001** | 6.8 |

*The effects in bold are significant at P < 0.05.*

**TABLE S2 Continued**

| **Parameter** | **Source of variation** | ***df*** | **Sum of squares** | **Mean squares** | ***F*** | ***P*** | **Variance explained (%)** |
| --- | --- | --- | --- | --- | --- | --- | --- |
| Bacteria | Drug (D) | 3 | 0.7 | 0.2 | 6.1 | ***P* < 0.001** | 0.9 |
|  | Concentration (C) | 2 | 3.7 | 1.8 | 50.9 | ***P* < 0.001** | 5.1 |
|  | Time (T) | 4 | 17.0 | 4.3 | 117.7 | ***P* < 0.001** | 23.6 |
|  | D × C | 6 | 1.1 | 0.2 | 5.0 | ***P* < 0.001** | 1.5 |
|  | D × T | 12 | 8.2 | 0.7 | 18.8 | ***P* < 0.001** | 11.3 |
|  | C × T | 8 | 27.2 | 3.4 | 94.0 | ***P* < 0.001** | 37.6 |
|  | D × C × T | 24 | 10.1 | 0.4 | 11.7 | ***P* < 0.001** | 14.0 |
| Fungi | Drug (D) | 3 | 1.6 | 0.5 | 13.7 | ***P* < 0.001** | 2.6 |
|  | Concentration (C) | 2 | 9.4 | 4.7 | 123.4 | ***P* < 0.001** | 15.3 |
|  | Time (T) | 4 | 21.2 | 5.3 | 139.9 | ***P* < 0.001** | 34.7 |
|  | D × C | 6 | 1.4 | 0.2 | 6.2 | ***P* < 0.001** | 2.3 |
|  | D × T | 12 | 2.3 | 0.2 | 5.0 | ***P* < 0.001** | 3.7 |
|  | C × T | 8 | 14.6 | 1.8 | 48.0 | ***P* < 0.001** | 23.8 |
|  | D × C × T | 24 | 6.2 | 0.3 | 6.8 | ***P* < 0.001** | 10.1 |

*The effects in bold are significant at P < 0.05.*
