# Supplementary material for: Variable Effects of Non-steroidal Anti-inflammatory Drugs (NSAIDs) on Selected Biochemical Processes Mediated by Soil Microorganisms
Source: Front Microbiol. 2016 Dec 5;7:1969. doi: 10.3389/fmicb.2016.01969 (PMC5147054; doi:10.3389/fmicb.2016.01969)
Supplement: Supplementary file 3 [file Table_3.DOC]

**TABLE S3 Results of a two-way MANOVA for the PC1 and PC2 based on the data for each sampling day.**

| **PC score** | **Day** | **Source of variation** | ***df*** | **Sum of squares** | **Mean squares** | ***F*** | ***P*** | **Variance explained (%)** |
| --- | --- | --- | --- | --- | --- | --- | --- | --- |
| PC1 | 1 | Drug (D) | 3 | 42.4 | 14.1 | 39.6 | ***P* < 0.001** | 20.0 |
|  |  | Concentration (C) | 2 | 119.2 | 59.6 | 167.3 | ***P* < 0.001** | 56.4 |
|  |  | D × C | 6 | 41.4 | 6.9 | 19.4 | ***P* < 0.001** | 19.6 |
|  | 15 | Drug (D) | 3 | 39.8 | 13.3 | 97.9 | ***P* < 0.001** | 33.9 |
|  |  | Concentration (C) | 2 | 26.1 | 13.0 | 96.1 | ***P* < 0.001** | 22.2 |
|  |  | D × C | 6 | 48.1 | 8.0 | 59.1 | ***P* < 0.001** | 41.0 |
|  | 30 | Drug (D) | 3 | 10.5 | 3.5 | 36.5 | ***P* < 0.001** | 5.4 |
|  |  | Concentration (C) | 2 | 176.0 | 88.0 | 917.6 | ***P* < 0.001** | 89.7 |
|  |  | D × C | 6 | 7.4 | 1.2 | 12.8 | ***P* < 0.001** | 3.8 |
|  | 60 | Drug (D) | 3 | 6.1 | 2.0 | 7.1 | ***P* = 0.001** | 3.3 |
|  |  | Concentration (C) | 2 | 161.7 | 80.9 | 286.3 | ***P* < 0.001** | 87.2 |
|  |  | D × C | 6 | 10.9 | 1.8 | 6.4 | ***P* < 0.001** | 5.9 |
|  | 90 | Drug (D) | 3 | 12.1 | 4.0 | 6.1 | ***P* < 0.005** | 9.5 |
|  |  | Concentration (C) | 2 | 82.6 | 41.3 | 62.4 | ***P* < 0.001** | 64.9 |
|  |  | D × C | 6 | 16.7 | 2.8 | 4.2 | ***P* < 0.005** | 13.2 |
| PC2 | 1 | Drug (D) | 3 | 1.7 | 0.6 | 1.9 | *P* = 0.143 | 10.7 |
|  |  | Concentration (C) | 2 | 3.9 | 1.9 | 6.9 | ***P* < 0.005** | 24.7 |
|  |  | D × C | 6 | 3.5 | 0.6 | 1.9 | *P* = 0.106 | 21.5 |
|  | 15 | Drug (D) | 3 | 5.3 | 1.8 | 9.0 | ***P* < 0.001** | 7.4 |
|  |  | Concentration (C) | 2 | 42.8 | 21.4 | 109.8 | ***P* < 0.001** | 59.9 |
|  |  | D × C | 6 | 18.7 | 3.1 | 16.0 | ***P* < 0.001** | 26.2 |
|  | 30 | Drug (D) | 3 | 14.7 | 4.9 | 15.7 | ***P* < 0.001** | 32.7 |
|  |  | Concentration (C) | 2 | 6.1 | 3.1 | 9.8 | ***P* < 0.001** | 13.6 |
|  |  | D × C | 6 | 16.6 | 2.8 | 8.8 | ***P* < 0.001** | 36.9 |
|  | 60 | Drug (D) | 3 | 4.4 | 1.5 | 1.7 | *P* = 0.189 | 15.3 |
|  |  | Concentration (C) | 2 | 0.2 | 0.1 | 0.1 | *P* = 0.875 | 0.8 |
|  |  | D × C | 6 | 3.8 | 0.6 | 0.7 | *P* = 0.629 | 12.9 |
|  | 90 | Drug (D) | 3 | 8.6 | 2.9 | 7.5 | ***P* = 0.001** | 17.3 |
|  |  | Concentration (C) | 2 | 14.7 | 7.3 | 19.4 | ***P* < 0.001** | 29.7 |
|  |  | D × C | 6 | 17.1 | 2.9 | 7.5 | ***P* < 0.001** | 34.6 |

*The effects in bold are significant at P < 0.05.*
