# Supplementary material for: Variable Effects of Non-steroidal Anti-inflammatory Drugs (NSAIDs) on Selected Biochemical Processes Mediated by Soil Microorganisms
Source: Front Microbiol. 2016 Dec 5;7:1969. doi: 10.3389/fmicb.2016.01969 (PMC5147054; doi:10.3389/fmicb.2016.01969)
Supplement: Supplementary file 4 [file Table_4.DOC]

**TABLE S4 Results of a two-way MANOVA for the PC1 and PC2 based on the data for each drug.**

| **PC score** | **Drug** | **Source of variation** | ***df*** | **Sum of squares** | **Mean squares** | ***F*** | ***P*** | **Variance explained (%)** |
| --- | --- | --- | --- | --- | --- | --- | --- | --- |
| PC1 | DCF | Time (T) | 4 | 17.7 | 4.4 | 14.5 | ***P* < 0.001** | 16.2 |
|  |  | Concentration (C) | 2 | 54.4 | 27.2 | 89.1 | ***P* < 0.001** | 49.8 |
|  |  | T × C | 8 | 28.1 | 3.5 | 11.5 | ***P* < 0.001** | 25.7 |
|  | NPX | Time (T) | 4 | 32.9 | 8.2 | 47.5 | ***P* < 0.001** | 19.2 |
|  |  | Concentration (C) | 2 | 72.1 | 36.1 | 208.5 | ***P* < 0.001** | 42.2 |
|  |  | T × C | 8 | 60.8 | 7.6 | 43.9 | ***P* < 0.001** | 35.6 |
|  | IBF | Time (T) | 4 | 16.0 | 4.0 | 22.9 | ***P* < 0.001** | 12.9 |
|  |  | Concentration (C) | 2 | 62.9 | 31.5 | 180.0 | ***P* < 0.001** | 50.9 |
|  |  | T × C | 8 | 39.2 | 4.9 | 28.1 | ***P* < 0.001** | 31.8 |
|  | KTP | Time (T) | 4 | 10.0 | 2.5 | 13.6 | ***P* < 0.001** | 7.6 |
|  |  | Concentration (C) | 2 | 29.1 | 14.5 | 78.9 | ***P* < 0.001** | 21.9 |
|  |  | T × C | 8 | 87.9 | 10.9 | 59.7 | ***P* < 0.001** | 66.3 |
| PC2 | DCF | Time (T) | 4 | 51.4 | 12.9 | 25.4 | ***P* < 0.001** | 65.1 |
|  |  | Concentration (C) | 2 | 3.1 | 1.6 | 3.1 | *P* = 0.060 | 3.9 |
|  |  | T × C | 8 | 9.2 | 1.2 | 2.3 | ***P* < 0.050** | 11.7 |
|  | NPX | Time (T) | 4 | 25.1 | 6.3 | 16.5 | ***P* < 0.001** | 45.3 |
|  |  | Concentration (C) | 2 | 9.7 | 4.9 | 12.8 | ***P* < 0.001** | 17.5 |
|  |  | T × C | 8 | 9.2 | 1.2 | 3. | ***P* = 0.013** | 16.7 |
|  | IBF | Time (T) | 4 | 12.1 | 3.0 | 9.1 | ***P* < 0.001** | 14.5 |
|  |  | Concentration (C) | 2 | 24.3 | 12.2 | 36.6 | ***P* < 0.001** | 29.4 |
|  |  | T × C | 8 | 36.5 | 4.6 | 13.8 | ***P* < 0.001** | 44.1 |
|  | KTP | Time (T) | 4 | 31.9 | 7.9 | 45.2 | ***P* < 0.001** | 28.6 |
|  |  | Concentration (C) | 2 | 32.3 | 16.1 | 91.3 | ***P* < 0.001** | 28.9 |
|  |  | T × C | 8 | 41.9 | 5.3 | 29.7 | ***P* < 0.001** | 37.7 |

*DCF, diclofenac; NPX, naproxen; IBF, ibuprofen; KTP, ketoprofen. The effects in bold are significant at P < 0.05.*
